# Immune checkpoint inhibitors in cancer therapy: Review of orofacial adverse events and role of the oral healthcare provider

**DOI:** 10.3389/froh.2022.968157

**Published:** 2022-08-11

**Authors:** Brittany A. Klein, Muhammad Ali Shazib, Alessandro Villa, Fábio de Abreu Alves, Piamkamon Vacharotayangul, Stephen Sonis, Stefano Fedele, Nathaniel S. Treister

**Affiliations:** ^1^Division of Oral Medicine and Dentistry, Brigham and Women's Hospital, Boston, MA, United States; ^2^Department of Oral Medicine, Infection, and Immunity, Harvard School of Dental Medicine, Boston, MA, United States; ^3^Division of Surgical and Specialty Care, Workman School of Dental Medicine, High Point University, High Point, NC, United States; ^4^Department of Orofacial Science, University of California San Francisco School of Dentistry, San Francisco, CA, United States; ^5^A.C. Camargo Cancer Center, São Paulo, Brazil; ^6^University of São Paulo School of Dentistry, São Paulo, Brazil; ^7^Department of Clinical Research, Eastman Dental Institute, University College London, London, United Kingdom; ^8^National Institute for Health and Care Research (NIHR) University College London Hospitals Biomedical Research Center, London, United Kingdom

**Keywords:** cancer, immunotherapy, oral medicine, oral pathology, toxicity

## Abstract

Immune checkpoint inhibitors (ICIs) are a revolutionary class of antineoplastic therapy that restore anti-tumor immunity. Consequences of this enhanced immune response include a multitude of immune related adverse events (irAEs) that can affect any body system, including the mouth. Orofacial irAEs reproduce features of numerous immune-mediated conditions, including oral lichen planus, mucous membrane pemphigoid, and Sjögren syndrome, among others. The aim of this review is to summarize known orofacial irAEs and to familiarize oral healthcare providers with how to identify and manage these toxicities as part of the care team for patients treated with ICIs.

## Introduction

Cytotoxic T cell lymphocyte-associated antigen (CTLA-4) and programmed cell death 1 (PD-1) and its ligand, programmed cell death 1 ligand (PD-L1), represent immune checkpoint pathways that downregulate T cell activation to promote peripheral tolerance. These pathways can be exploited by tumor cells to promote immune evasion [1]. Immune checkpoint inhibitors (ICIs) block these receptor-ligand relationships, thereby restoring and activating anti-tumor immunity [2]. ICIs have dramatically improved outcomes in an extensive and growing list of solid (e.g., melanoma, lung, head and neck, colorectal) and hematologic (e.g., Hodgkin and non-Hodgkin lymphoma, multiple myeloma) malignancies, both in metastatic disease and, increasingly, in earlier stages and (neo)adjuvant settings [3, 4]. There are currently eight Food and Drug Administration (FDA)-approved agents targeting CTLA-4 (ipilimumab), PD-1 (pembrolizumab, nivolumab, cemiplimab, dostarlimab), and PD-L1 (atezolizumab, durvalumab, avelumab) [4, 5].

ICIs modulate endogenous regulatory immune mechanisms to enhance immune system activation and mount a successful immune response against tumor cells [1]. However, this activation occurs broadly, is non-specific, and can lead to a wide variety of immune-related adverse events (irAEs) [6, 7]. These irAEs can affect any body system at any time during the course of or following treatment, though they most commonly present within the first months of therapy [3, 8]. The skin tends to be the earliest (i.e., between 2 and 12 weeks after initiating treatment) and the most frequently affected site [9]. These events are not uncommon: 60% of patients treated with an anti-CTLA-4 antibody, nearly 30% of patients treated with an anti-PD-1 or anti-PD-L1 antibody, and as many as 90% treated with a combination of a CTLA-4 and PD-1 or PD-L1 inhibitor will experience one or more irAEs [8]. IrAEs can be acute and reversible, but may also be chronic and/or permanent toxicities (i.e., endocrine and rheumatologic irAEs) in as many as 43% of patients [4, 10]. Severity of irAEs can vary and there are a number of clinical grading systems that have been proposed; the most ubiquitous in the literature is the National Cancer Institute (NCI) Common Terminology Criteria for Adverse Events (CTCAE) version 5—a five-point scale from grade 1 (mild) to grade 5 (death) [11]. An objective and widely adopted grading system is a crucial tool to inform management of ICI therapy. As a general rule, ICIs are temporarily held in the setting of a grade 3 (severe) irAE and permanently discontinued for any grade 4 (life-threatening) irAE [12]. Corticosteroids are a mainstay of treatment for irAEs, though steroid-sparing, reaction-specific regimens (e.g., mycophenolate mofetil, infliximab, hydroxychloroquine) are being increasingly utilized [3, 6, 12]. While irAEs can be associated with significant morbidity and even mortality, there is emerging evidence that they are a positive predictor of clinical outcomes [13].

Oral mucosal and salivary gland irAEs have been inconsistently reported and classified, so the prevalence is not clearly defined, though the incidence may be as high as seven percent [14, 15]. They can occur with or without cutaneous or systemic manifestations [9]. This review summarizes current knowledge on orofacial irAEs and suggests a pragmatic approach to their identification and management by the oral healthcare provider (OHP).

## History and examination for the oral healthcare provider

For cancer patients planned to initiate immunotherapy, OHPs should be aware of any existing immune-mediated conditions. ICI therapy may exacerbate pre-existing immune-mediated conditions, and it is important to be able to distinguish a *de novo* irAE from an exacerbation of an underlying disease process [1]. Patients should be made aware of this risk and be encouraged to notify their care team about worsening or new symptoms. A comprehensive oncologic history that includes any past or concurrent treatments should be obtained, as ICI therapy may be given in combination with cytotoxic chemotherapy, radiation, other targeted therapies, or even supportive care measures that can introduce additional risk factors/concomitant side effects (e.g., bone marrow suppression, osteonecrosis of the jaw) [3].

**Figure 1:**
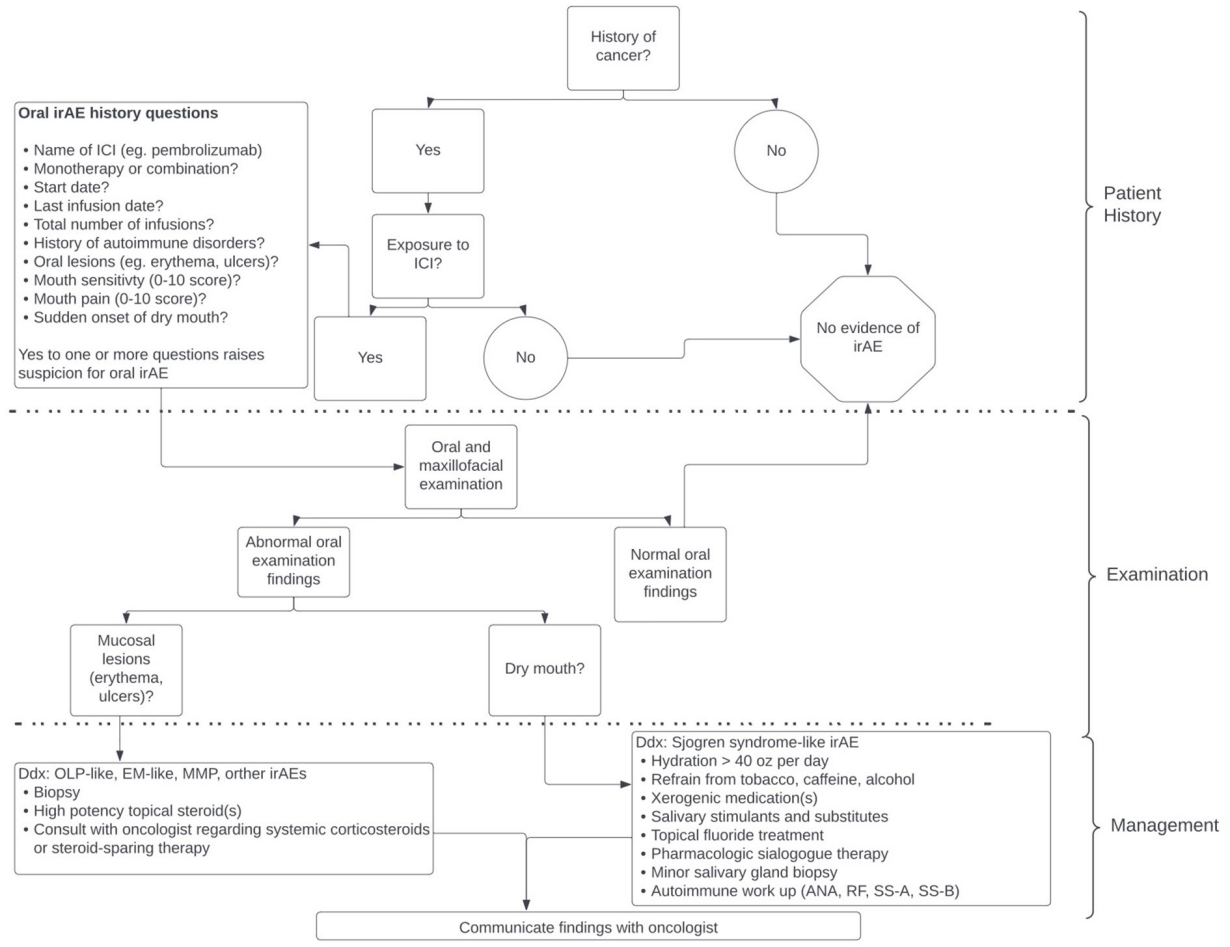
Approach to identifying patients with immune-related adverse events (irAEs) and overview of management.

Workup of a patient with a suspected oral irAE should begin with a thorough medical history and history of present illness (Figure 1). Inquire about the onset (sudden or gradual), duration (days, weeks, or months), nature (pain, sensitivity, difficulty eating/swallowing, etc.) and severity of symptoms (visual analog scale (VAS) pain/sensitivity score), sites affected (including extraoral sites), and any other irAEs they have experienced [16]. A review of systems (ROS) should also be performed as this may identify other extra-oral irAEs. Any positive findings, whether identified by patient report, ROS, or physical examination (for cutaneous lesions in particular) should be communicated to the patient and their oncologist.

When conducting a head and neck intra-oral and extra-oral examination in this patient population, general principles apply [17]. Each site should be examined methodically and an attempt should be made to assess salivary gland function by expressing saliva from the major salivary gland ducts. All findings should be documented and thoroughly described, including location, number, size (with measurements if possible), color, and texture of any mucosal abnormality (including that of saliva). Clinical photographs can be a helpful tool to monitor oral mucosal lesion progression (or resolution) and to communicate with other members of the patient's care team.

## Oral mucosal irAEs

**Figure 2:**
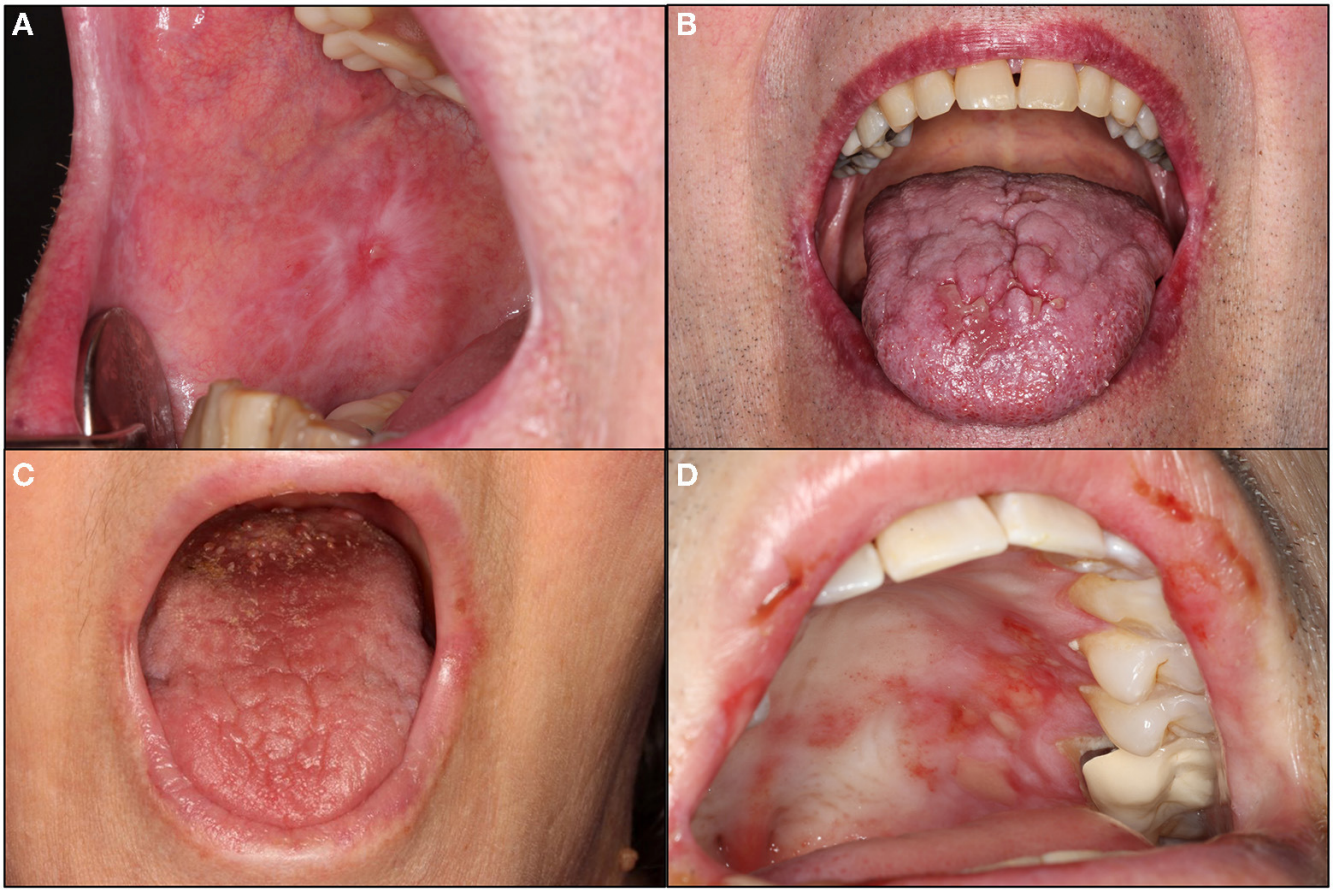
Oral mucosal and salivary immune related adverse events (irAEs). **(A,B)** demonstrate oral lichen planus-like irAEs characterized by white striations of the right buccal mucosa with a central pinpoint ulceration and surrounding erythema **(A)** and generalized erythema of the upper and lower lip vermilion with atrophy, white changes, and coalescing ulceration of the midline anterior dorsal tongue with surrounding erythema (HSV-negative; **B**). **(C)** demonstrates a Sjögren syndrome-like irAE with desiccated oral mucosa and loss of filiform papillae of the dorsal tongue. **(D)** demonstrates a mucous membrane pemphigoid irAE characterized by scattered ulcerations of the hard palatal mucosa and upper lip mucosa with significant surrounding erythema (HSV-negative).

Oral mucosal irAEs typically mimic or recapitulate the pathogenesis and clinical features of a range of well-defined immune-mediated mucocutaneous disorders, including oral lichen planus (OLP), mucous membrane pemphigoid (MMP)/bullous pemphigoid (BP), erythema multiforme (EM), and Stevens-Johnson syndrome (SJS)/toxic epidermal necrolysis (TEN) (Figure 2) [3, 16, 18]. These reactions may also present with overlapping features of more than one condition [16].

OLP-like irAEs present with white striations, erythema, and/or ulcerations of the oral mucosa, particularly the ventral tongue and buccal mucosa, ranging from asymptomatic to severely painful [19, 20]. Cutaneous LP-like irAEs are one of the most common dermatologic irAEs, present in between 0.5 and 6% of patients, so many patients with oral lesions will also have skin involvement [9]. Autoimmune cutaneous blistering disorders are also common—affecting 1% of patients treated with ICIs [21]. BP only rarely includes oral lesions, whereas MMP often exclusively affects the oral mucosa, presenting with desquamative gingivitis, erosions, ulcers, and/or intact bullae [21–24]. EM-like irAEs typically present with targetoid skin lesions and, intraorally, multiple irregularly shaped erosions and ulcers with hemorrhagic crusting of the lips; cases involving exclusively the oral mucosa, while controversial, have also been described [25–27]. SJS and TEN-like irAEs represent severe and, in the case of TEN especially, life-threatening mucocutaneous reactions with oral lesions resembling that of EM, including multiple, large, irregularly-shaped, ulcerations/erosions and hemorrhagic crusting of the lips [18, 28]. There have additionally been cases reported of systemic lupus erythematous and scleroderma with oral involvement, as well acute oral GVHD reactivation and linear IgA disease [18, 29–31]. These irAEs are rare, underrepresented, and/or poorly characterized, underscoring the fact this is an evolving area of study.

Diagnosis of oral mucosal irAEs can be confirmed based on histopathology and/or immunofluorescent studies, though in some cases, clinical diagnosis may be sufficient. Biopsies should ideally be obtained prior to initiating treatment of the irAE. If a vesiculobullous condition is suspected, specimens should be submitted for both histopathologic analysis and direct immunofluorescence (DIF). In the appropriate clinical context, indirect immunofluorescence (IIF) and/or enzyme-linked immunosorbent assay (ELISA) can also be considered.

Histopathologic features of oral mucosal irAEs, much like the clinical presentation, may mimic the condition or can have absent, overlapping, or non-specific findings [20, 32]. OLP-like irAEs are characterized by interface mucositis and may exhibit a dense lymphocytic band, basal vacuolar changes, spongiosis, and/or subepithelial clefting [20, 32]. On DIF, a non-specific patchy deposition of fibrinogen will be observed at the basement membrane zone without specific reactivity to immunoglobulins [20]. ELISA is negative to antidesmoglein-1 (Dsg-1), antidesmogelin-3 (Dsg3), and anti-BP180 (BP180) antibodies [33]. There are no published oral mucosal histopathology findings in BP irAEs, but skin immunofluorescence studies reveal linear deposition of IgG and C3 at the dermo-epidermal junction and positive serologic titers to BP180 [21, 34]. Features of MMP include subepithelial clefting with preservation of the basal layer of epithelium and a mixed perivascular inflammatory infiltrate on histopathology and linear deposits reactive to IgG, IgA, and C3 on DIF [23, 35]. Approximately half of MMP irAE cases are positive for BP180 [23, 35]. Histopathologic and immunofluorescent findings in EM-like irAEs are non-specific and include a mixed inflammatory infiltrate and no specific reactivity [36]. In such cases, the absence of findings is itself revealing. Oral lesions of SJS and TEN will similarly reveal non-specific ulceration and inflammation [37]. Among any of these conditions, overlapping histopathologic features are possible.

We have previously published suggested grading criteria based upon symptom severity and impact on oral alimentation and accompanying management guidelines for oral mucosal and salivary irAEs [16]. The grading criteria for oral mucosal irAEs draw from several established guidelines for irAEs [i.e., CTCAE, American Society of Clinical Oncology (ASCO), National comprehensive Cancer Network (NCCN), and Society for Immunotherapy of Cancer (SITC)] and range from grade 1 (asymptomatic or mildly symptomatic) to grade 4 (severely painful oral lesions making oral alimentation impossible). In line with these established guidelines, we generally recommend that systemic steroids be considered for any grade ≥2 irAE and the ICI be temporarily held for any grade ≥3 toxicity with consideration of permanent discontinuation for any grade 4 toxicity.

Integral to the management approach of oral mucosal irAEs is early and aggressive intervention with high-potency topical steroids [15]. Solution formulations work well for multifocal and/or hard to reach lesions, and gels can be applied to focal lesions with gauze or a cotton tip applicator. Adequate contact time is critical to ensure maximum efficacy (e.g., hold solution or leave gauze in place for 5 min). If lesions are severe at presentation, fail to respond to, or progress while using topical steroids, systemic treatment is indicated, generally with oral prednisone at a dose of 1 mg/kg followed by a slow taper to avoid flares [6]. The plan to initiate corticosteroids should be coordinated with the patient's oncologist, who may make the decision to hold ICI therapy until lesions improve or resolve. Steroid-sparing immunosuppressive agents can also be considered, such as doxycycline, mycophenolate mofetil, acitretin, infliximab, dupilumab, or IVIG [6]. EM-like oral mucosal manifestations may be managed with topical corticosteroids (plus or minus systemic prednisone), whereas mucocutaneous manifestations of SJS TEN-like irAEs require aggressive, multidisciplinary inpatient management [6]. If the ICI is rechallenged or another is initiated, patients should be followed closely by their oral healthcare provider to identify recurrence, flares, or new irAEs.

## Salivary gland irAEs

IrAEs affecting the salivary glands occur, as with other irAEs, along a spectrum of severity and are generally referred to as sicca syndrome or Sjögren syndrome (SS)-like, reflecting the largely shared symptoms, clinical features, and treatment approaches to these entities (Figure 2) [38, 39]. While clinically similar to Sjögren-syndrome, there is some histopathologic evidence that SS-like irAEs may be mediated primarily through autoreactive T cells rather than the B cells classic of SS [40]. There are, however, cases that meet the diagnostic criteria for SS, suggesting they are clinically indistinguishable from SS [38].

Xerostomia is reported by 0.4–7% of patients treated with ICIs, though some of these may be attributable to other causes (e.g., polypharmacy, dehydration) [14, 38]. Patients with a true salivary irAE typically present with acute onset of severe dry mouth and hyposalivation, with or without dry eyes [38, 39]. A thorough diagnostic workup may include measurement of whole unstimulated salivary flow rate (WUSF), minor salivary gland biopsy (i.e., from the labial mucosa), and serology (ANA, RF, anti-Ro, anti-La). That said, clinical judgement should be employed to weigh the utility of such tests. For instance, clinical examination may be sufficient to assess for hyposalivation based on any of the following: visibly desiccated mucosa, lack of floor of mouth pooling, inability to express saliva from the parotid or submandibular gland ducts, mirror or glove sticking to the mucosa, or qualitative changes to the saliva (i.e., frothy, sticky, or ropey). In this patient population, serology is positive in only a minority of cases [38, 39]. Similarly, histopathology of minor salivary gland biopsies in SS-like irAEs variably demonstrate the focal sialadenitis characteristic of SS; half of cases demonstrate non-specific chronic sialadenitis with a focus score of zero [38, 39].

As with oral mucosal irAEs, we have previously proposed a set of grading criteria and management guidelines for salivary irAEs informed by CTCAE, European League Against Rheumatism (EULAR) guidelines for the management of SS, and the guidelines published by Klein et al. [16] and Warner et al. [39]. In this case, the grading criteria range from grade 1, characterized by xerostomia without hyposalivation or impact on diet, to grade 3, which presents with hyposalivation so severe as to prevent adequate oral alimentation and/or systemic features of SS that impact the patient's ability to perform activities of daily living (ADLs). In a patient with a history of exposure to ICIs with a complaint of dry mouth, it is important to recognize other common etiologies of xerostomia/hyposalivation including dehydration, polypharmacy, and anxiety. Regardless of grade, symptomatic management is a cornerstone, including over the counter mouth moisturizers and saliva substitutes as well as prescription sialagogues (e.g., pilocarpine or cevimeline). Adequate hydration and avoidance of caffeine and smoking should also be encouraged. Once there is evidence of hyposalivation, topical fluoride supplementation should be prescribed to prevent caries [41]. If there is impact on diet and/or systemic features of SS, this should be communicated to the patient's oncologist who may elect to hold the ICI and/or involve a rheumatologist. Systemic treatment options for grades 2 and 3 include prednisone, hydroxychloroquine, or other disease modifying antirheumatic drugs (DMARDs) [3, 39].

## Other orofacial irAEs

There have been at least four reported cases of medication related osteonecrosis of the jaw (MRONJ) related to ICIs in patients with no prior or concurrent exposure to antiresorptive or anti-VEGF therapies [42–45]. Three cases occurred spontaneously, and one occurred following a dental extraction. All presented with pain (of varying degrees) and local swelling, with a sinus tract, exposed bone, or non-healing extraction site observed on initial examination [42–44]. Ultimately, all cases exhibited clinically exposed necrotic bone. Radiographic (e.g., panoramic radiograph or computed tomography) findings were variable among cases and included a moth-eaten trabecular pattern with bilateral mandibular fractures, maxillary sinusitis with osteolysis, and a non-healing extraction site [42, 44, 45]. Antibiotics (e.g., amoxicillin-clavulanate or amoxicillin/metronidazole) were prescribed in all four cases, and chlorhexidine rinses in three cases. Sequestrectomy was performed in three cases, followed by complete re-epithelization in two cases [43, 44]. For the case complicated by bilateral mandibular fractures, a total mandibulectomy with fibula reconstruction was performed; histopathology confirmed necrosis of the trabecular and cortical bone with fibrosis of the marrow space [42]. OHPs should be alert to the possibility of osteonecrosis as an irAE secondary to ICI therapy in addition to better known culprit medications (e.g., bisphosphonates, denosumab).

Dysgeusia has been reported as an irAE in 16 randomized controlled trials, with a pooled incidence of 4.9% [46]. A recent single center retrospective review estimated the incidence to be 3.6% [14]. Further studies are needed to characterize the features, clinical course, and management approach. OHPs should be aware of this as a possible explanation for taste changes in patients who have been treated with ICIs.

## Conclusion

ICIs have quickly become a mainstay of cancer therapy [3]. Thus, it is important for practitioners of all disciplines to recognize both their therapeutic mechanisms and adverse events (irAEs), which are distinct from conventional cytotoxic chemotherapy. OHPs can provide three key roles for patients who are initiating, actively being treated with, or who have been on an ICI: [1] identification of and supportive care for orofacial irAEs; [2] communication of orofacial and/or pertinent positive systemic findings to the oncologist; [3] continued routine dental treatment with emphasis on the maintenance of oral hygiene practices. With attention to each of these facets, OHPs can play a critical supportive role in the multidisciplinary oncology team for patients treated with ICIs.

## Author contributions

BK, MS, SF, and NT contributed to conceptualization. BK and MS contributed to writing (original draft and editing). AV, FA, PV, SS, SF, and NT contributed to reviewing and editing. All authors contributed to the article and approved the submitted version.

## Conflict of interest

The authors declare that the research was conducted in the absence of any commercial or financial relationships that could be construed as a potential conflict of interest.

## Publisher's note

All claims expressed in this article are solely those of the authors and do not necessarily represent those of their affiliated organizations, or those of the publisher, the editors and the reviewers. Any product that may be evaluated in this article, or claim that may be made by its manufacturer, is not guaranteed or endorsed by the publisher.
